# Effects of Aquatic Conditions on Trap Structure in the Submerged Form of *Drosera intermedia* Hayne

**DOI:** 10.3390/plants15132081

**Published:** 2026-07-03

**Authors:** Izabela Kozak, Krzysztof Banaś, Bartosz J. Płachno

**Affiliations:** 1 Institute of Botany, Faculty of Biology, Jagiellonian University in Kraków, 9 Gronostajowa St., 30-387 Kraków, Poland; 2 Department of Plant Ecology, Faculty of Biology, University of Gdansk, 59 Wita Stwosza St., 80-308 Gdańsk, Poland

**Keywords:** aquatic plants, carnivorous plants, *Drosera intermedia*, *Drosera* habitat, emergence, sundews, tentacle types, trap architecture

## Abstract

Carnivorous plants possess highly specialized trap structures that enable the acquisition of nutrients from captured animals. This study investigated whether the aquatic environment influences the architecture of glandular emergences (tentacles) in the submerged form of *Drosera intermedia*. Morphometric analyses were performed on tentacles from plants growing in sandy terrestrial habitats, emergent wetland habitats, and submerged aquatic habitats. Significant differences in tentacle morphology were detected among habitat types. Emergent plants generally developed the largest tentacles and tentacle heads, whereas submerged plants exhibited shorter marginal tentacles and smaller glandular heads. In contrast, the shape of both marginal and central tentacle heads remained relatively stable across habitats, indicating stronger developmental conservation of this trait. Nevertheless, the submerged form differed significantly in the morphology of central tentacle heads. These results demonstrate that aquatic conditions substantially modify the structure of carnivorous emergences, revealing pronounced phenotypic plasticity in the carnivorous syndrome of *D. intermedia*. The reduction in tentacle size under submerged conditions may reflect altered functional demands associated with prey capture, changes in mucilage effectiveness, and the mechanical constraints imposed by the aquatic environment. However, differences in light availability, nutrient status, and other habitat characteristics may also contribute to the observed patterns. At the same time, the persistence of glandular structures indicates the retention of the carnivorous apparatus despite prolonged submergence. This study highlights the remarkable developmental flexibility of *D. intermedia* and provides new evidence that trap morphology in carnivorous plants may be highly responsive to environmental conditions.

## 1. Introduction

Since the time of Charles Darwin [1], carnivorous plants have attracted the attention of scientists, the general public, and hobbyists alike [2,3,4,5]. Carnivorous plants, also referred to as insectivorous plants, are mixotrophic organisms that have evolved a complex carnivorous syndrome involving prey attraction and capture, the digestion of trapped organisms, and the subsequent absorption of nutrients derived from prey tissues [4,6,7,8,9]. However, carnivory is energetically costly because it requires substantial investment in trap production and is often associated with reduced photosynthetic efficiency. Consequently, carnivory is advantageous only under specific environmental conditions, particularly in habitats with sufficient light availability [10,11]. Under low-light conditions, carnivorous plants may lose their carnivorous characteristics, either failing to produce traps altogether or developing malformed trapping structures.

In species of the genus *Drosera* L. (sundews), the margins and adaxial surface of the leaf blade are covered by specialized emergences, commonly referred to as tentacles [1,12]. The most widespread tentacle type, designated as T0, consists of radially symmetrical glandular emergences composed of a slender stalk and a terminal glandular head [13,14]. These structures secrete sticky mucilage that attracts and immobilizes prey. Subsequently, the same glands release digestive enzymes and absorb nutrients liberated from prey tissues [4,15]. In addition to carrier-mediated uptake, nutrient acquisition in *Drosera* also involves endocytosis, enabling the absorption and intracellular digestion of intact proteins [15,16,17]. Several additional types of emergences have been described within the genus [13,14,18]. Particularly remarkable are the snap-tentacles of *Drosera glanduligera* Lehm., which can complete their bending movement within approximately 75 ms following mechanical stimulation [19]. Altogether, glandular emergences are essential structural and functional components of the carnivorous syndrome in *Drosera*.

**Figure 1:**
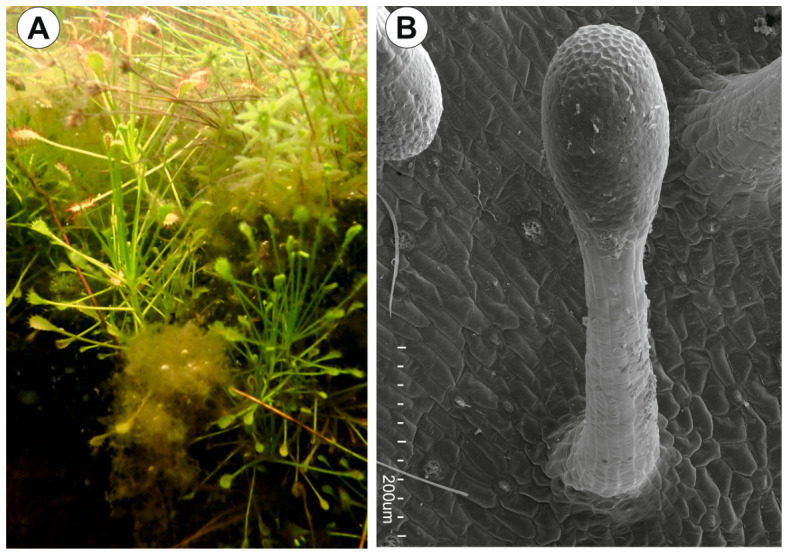
(**A**) A submerged growth form of *D. intermedia*. (**B**) A glandular emergence from *D. intermedia* leaf. Bar 200 µm.

Recently, it was demonstrated that some populations of *Drosera intermedia* Hayne (oblong-leaved sundew) develop a fully submerged growth form (Figure 1A) that is capable not only of surviving but also of growing underwater for many years [20]. According to Banaś et al. [20], submerged plants differ markedly from terrestrial and peatland forms in both plant architecture and photosynthetic performance. In this context, the present study aims to determine whether long-term growth under aquatic conditions affects the morphology and spatial distribution of glandular emergences in *D. intermedia*. Specifically, we tested the hypothesis that the submerged form of *D. intermedia* undergoes a partial functional reduction in carnivorous syndrome.

## 2. Results

All examined plants exhibited T0-type emergences on their leaves (Figure 1B).

### 2.1. Differences in the Lengths of Tentacles of Leaf Blades

**Figure 2:**
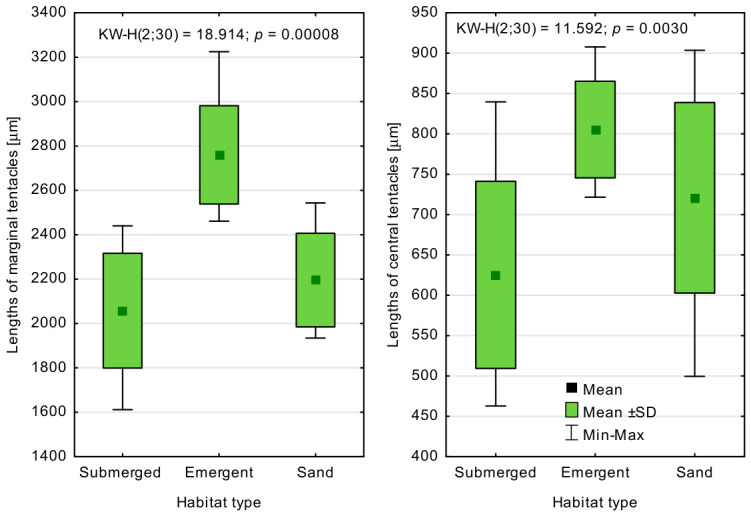
Length of tentacles on the marginal and central parts of leaf blades of *D. intermedia* depending on habitat type.

The length of the tentacles on the marginal parts of the leaf blades of *D. intermedia* varies among populations from different habitat types (Table 1). The submerged form is characterized by the shortest tentacle length (2057.827 ± 258.37 µm; Figure 2). Post hoc Dunn’s tests indicated significant pairwise differences between submerged and emergent habitat types (Table 2) on the marginal parts of the leaf blades. The mean values are highest in the emergent form.

The lengths of tentacles on the central parts of the leaf blades are more than three times shorter than those on the marginal parts. The submerged form also has the shortest tentacle length, but only the difference relative to the emergent form is statistically significant. Sundews growing as emergent plants are characterized by the longest tentacle lengths (Table 2; Figure 2), which are greater than those of both the submerged form (*p* = 0.000116) and the form growing on sand (*p* = 0.00315; Table 2).

### 2.2. Differences in the Shapes of Tentacle Heads

**Figure 3:**
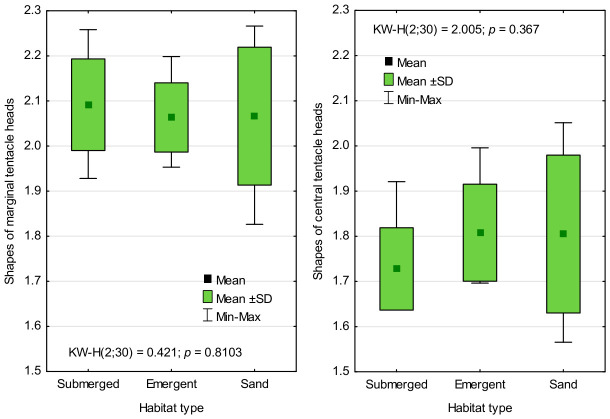
Variation in the shapes of tentacle heads on the margins and central parts of leaf blades of *D. intermedia* across habitat types.

The Kruskal–Wallis test does not indicate significant differences in the shapes of tentacle heads among habitat types (Figure 3). For the tentacles at the margins of the leaf blades and those in their central parts, the head shape values are very similar (Table 3).

### 2.3. Differences in the Dimensions of Tentacle Heads

#### 2.3.1. Tentacle Heads Lengths

**Figure 4:**
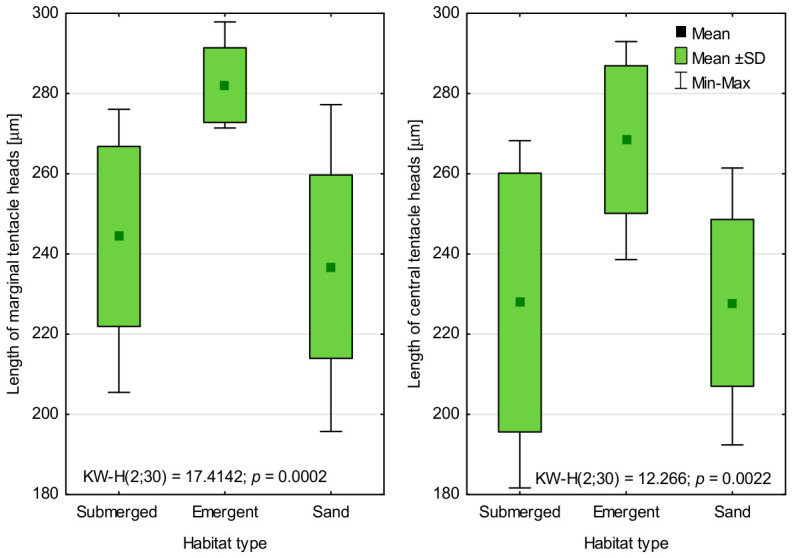
Mean length of tentacle heads on the marginal and central parts of leaf blades of *D. intermedia* across habitat types.

A Kruskal–Wallis test indicated significant differences in the length of tentacle heads among habitat types (Figure 4). This applies to tentacles found both at the margins of the leaf blade and in its central part. The highest mean values were recorded in emergent habitats (Table 4). Dunn post hoc tests showed significant differences between emergent and sandy and between emergent and submerged habitats. Sandy and submerged habitats did not differ significantly (Table 5).

#### 2.3.2. Tentacle Heads Widths

**Figure 5:**
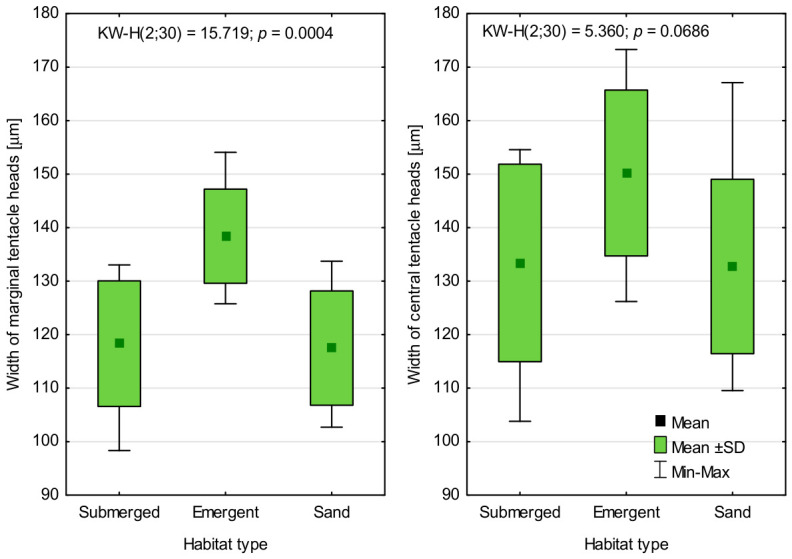
Widths of tentacle heads on the marginal and central parts of leaf blades of *D. intermedia* across habitat types.

Emergent habitats exhibited the greatest mean widths (Table 6; Figure 5). Significant differences in tentacle head width were also detected (Table 7), but only for marginal tentacle heads. Post hoc tests indicated significant differences between emergent and sandy and between emergent and submerged habitats, with no significant difference between sandy and submerged habitats (Table 7).

### 2.4. Morphological Traits and Habitat Characteristics

**Figure 6:**
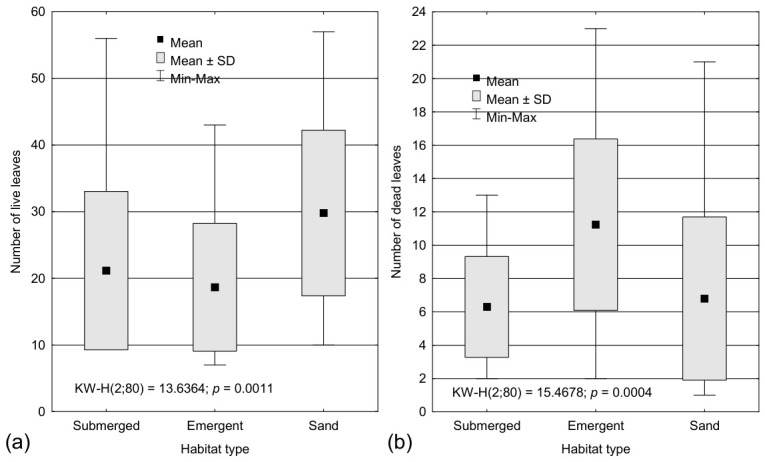
Number of live (**a**) and dead (**b**) leaves in *D. intermedia* across habitat types. Both traits differed significantly among habitats.

**Figure 7:**
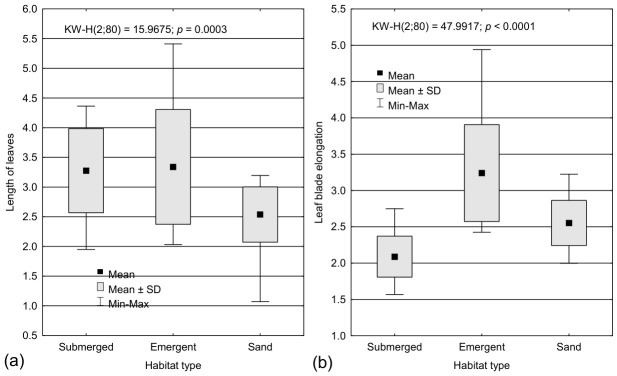

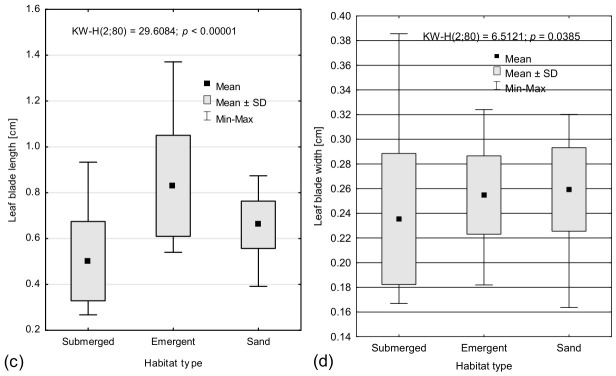
Variation in leaf morphological traits of *D. intermedia* across habiat types: (**a**) leaf length, (**b**) blade elongation, (**c**) blade length, and (**d**) blade width.

**Figure 8:**
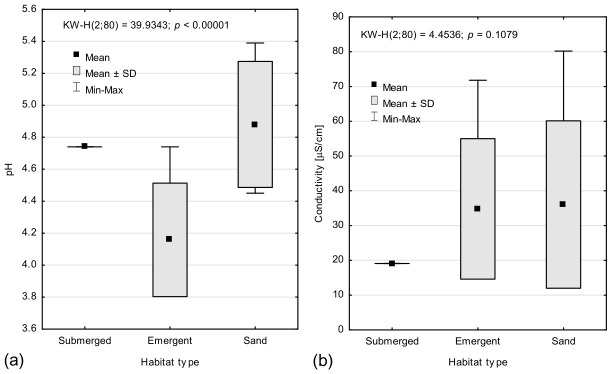

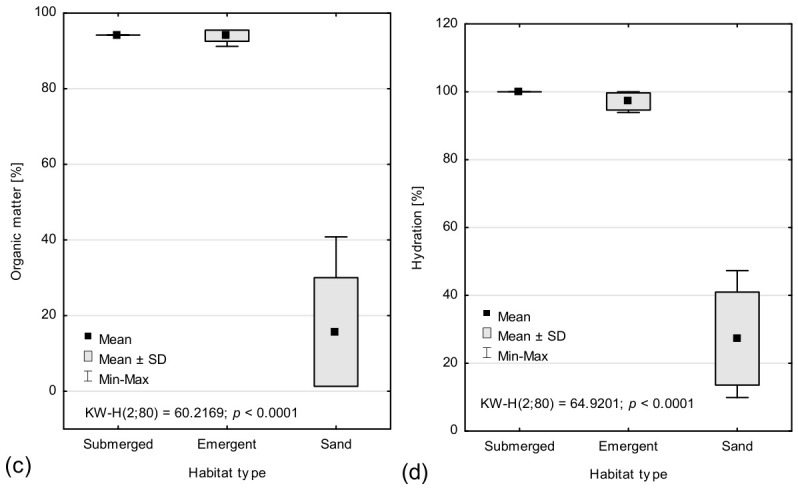
Environmental and physiological characteristics of *D. intermedia* habitats: (**a**) pH, (**b**) conductivity, (**c**) organic matter content, and (**d**) hydration.

**Figure 9:**
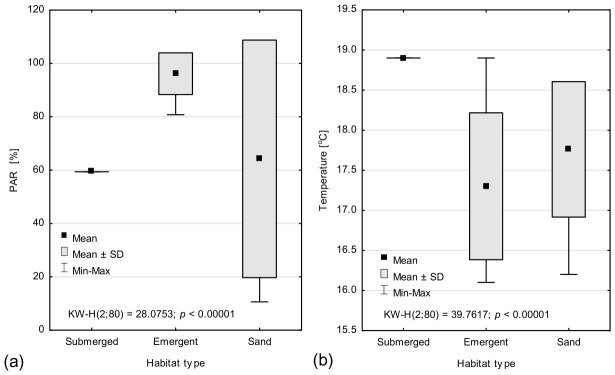
Differences in environmental conditions among *D. intermedia* habitats: (**a**) PAR and (**b**) temperature.

In morphological traits, the leaves of the sundew growing on sand stand out clearly (Table 8, Table 9 and Table 10, Figure 6, Figure 7, Figure 8 and Figure 9). They have the highest number of livie leaves in the rosette, more than both emergent plants (*p* = 0.001) and submerged plants (*p* = 0.03). Like emergent sundews, they have fewer dead leaves in the rosette than emergent sundews. However, they are distinguished by the shortest leaf length, which is shorter than those of both emergent individuals on peat bogs and those growing submerged (*p* = 0.002).

The elongation of the leaf blade varies considerably among the studied forms (*p* < 0.002); it is shortest in the submerged form, slightly greater in plants growing on sand, and greatest in the emergent form (Figure 7). The length of the leaf blade also varies very markedly, as shown in Figure 7; it is shortest in the submerged form and longest in the emergent form. In contrast, the width of the leaf blade in the submerged form is smaller only than that of plants growing on sand.

The habitats occupied by the sundews studied differ in many characteristics. Emergent forms occur on substrates with a lower pH than submerged and sand forms, which also receive the most light (PAR is highest; Figure 8 and Figure 9). The submerged form occupies habitats with a significantly higher temperature than the other forms, whilst the sand form occupies habitats that are the least hydrated and the least rich in organic matter, and, of course, the richest in mineral matter.

**Figure 10:**
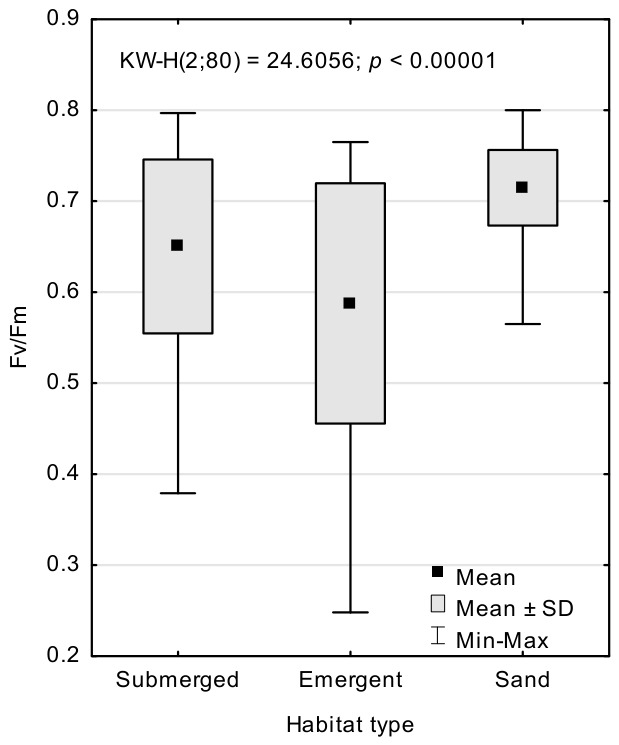
Differences in Fv/Fm ratio among *D. intermedia* habitats.

The highest Fv/Fm ratio was observed in the sand form; it is higher than in both the submerged and emergent forms (Figure 10). Interestingly, the ratio is lowest in the emergent form (Table 9), which may indicate that these sundews are in the poorest condition, despite occupying habitats most typical of *D. intermedia*.

## 3. Discussion

The present study demonstrates that the aquatic environment strongly influences the architecture of carnivorous traps in the submerged form of *Drosera intermedia*. Although these differences are consistent with an effect of submergence, the studied habitats also differed in several other environmental parameters, including light availability, substrate characteristics, water content, pH, and temperature. Therefore, the observed variation likely reflects the combined influence of multiple environmental factors rather than submergence alone. Significant differences were detected in tentacle length, tentacle head dimensions, and the shape of central tentacle heads among plants growing in sandy, emergent, and submerged habitats. The obtained results indicate that the submerged form does not merely represent a phenotypic variant with altered growth habit but exhibits substantial modifications in trap morphology associated with the aquatic environment.

The most conspicuous pattern concerns tentacle elongation. Tentacles on both the central and marginal parts of the leaf blade differed significantly among habitat types, with emergent plants generally exhibiting the longest tentacles, whereas submerged plants developed the shortest marginal tentacles. This reduction in tentacle length in submerged forms may reflect the altered mechanical and ecological conditions under water. However, differences in light regime, nutrient availability, and other habitat characteristics may also contribute to the observed pattern. Consequently, the present data do not allow the relative importance of individual environmental variables to be disentangled. In terrestrial and emergent habitats, long tentacles increase the effective prey-capture surface and facilitate interception of mobile arthropods. Underwater, however, elongated emergences may become mechanically disadvantageous because water resistance substantially alters movement efficiency and increases energetic costs. Similar environmentally induced modifications or even loss of carnivorous structures have been described in other carnivorous plants, where trap morphology is tightly linked to habitat conditions. One example is the production of phyllodia by *Sarracenia*, which are more efficient at photosynthesis [21]. Another example is the African liana *Triphyophyllum peltatum* Airy Shaw, which exhibits seasonal carnivory [22]. It produces trap leaves only when there is a phosphorus deficiency in the soil [23]. Fukushima et al. [24] demonstrated that in *Cephalotus follicularis* Labill., the formation of the photosynthetic leaf or insect-catching trap is regulated by two co-varying environmental signals: temperature and photoperiod. Whitewoods et al. [25] showed that the simple shifts in gene expression may cause the formation of a traps in *Utricularia gibba* L.

The observed reduction in tentacle head size in submerged plants is also ecologically meaningful. Emergent plants possessed significantly larger tentacle heads than submerged or sandy forms, both in the central region and on leaf margins. Since glandular heads are responsible for mucilage secretion, digestive enzyme production, and nutrient absorption, these results suggest that submerged plants may exhibit reduced investment in typical aerial carnivorous functions. In aquatic conditions, mucilage-based trapping becomes less efficient because secreted mucilage disperses in water and loses adhesive properties. Consequently, selective pressure maintaining large glandular heads may be weakened.

Interestingly, the shape of tentacle heads showed different responses depending on their position on the leaf blade. Significant differences occurred in the central tentacles, particularly between submerged and non-submerged forms, whereas marginal tentacle head shape remained remarkably stable among habitats. This pattern suggests that different functional modules within the trap exhibit distinct levels of developmental plasticity. Marginal tentacles may be under stronger structural or functional constraints because they participate directly in prey interception and leaf-edge architecture. In contrast, central tentacles appear more evolutionarily flexible and responsive to environmental conditions.

The results may support the hypothesis that the submerged form of *D. intermedia* undergoes partial functional reduction in the carnivorous syndrome. This interpretation should be treated cautiously because the present study does not permit discrimination between the effects of submergence itself and those of other environmental variables that differed among habitats. However, further research is needed to prove this conclusively. Carnivory is energetically expensive because it requires the production of specialized emergences, digestive secretions, and absorptive tissues. Underwater conditions likely alter prey availability, prey type, and nutrient dynamics, this may potentially reduce the adaptive advantage of maintaining fully developed in aerial traps. This interpretation is consistent with the cost–benefit model of carnivory proposed by Givnish and co-authors [10,11], who emphasized that carnivory is only advantageous under specific combinations of nutrient limitation and sufficient light availability.

At the same time, the submerged form clearly retains carnivorous structures rather than completely eliminating them. This indicates that the observed modifications represent ecological adjustment rather than evolutionary loss of carnivory. Such phenotypic plasticity may be particularly advantageous in unstable wetland habitats where water level fluctuations periodically shift plants between terrestrial, emergent, and submerged conditions, while simultaneously altering other environmental parameters such as irradiance, substrate moisture, and nutrient availability. The submerged form occurs under conditions that are difficult and extreme for *D. intermedia*, similar to the sand-dwelling form, as confirmed by the close similarity of many of the characteristics we studied, which differ greatly from the typical emergent form occupying the habitat typical of this species. Our research contributes to a broader understanding of how the environment (and water in particular) influences the architecture and physiology of plants in the genus *Drosera* [20,26].

The present study also has broader implications for understanding the evolution of trap diversity in Droseraceae. Previous studies mainly focused on interspecific differences in trap architecture and physiology, whereas the current results demonstrate that substantial variation may also occur within a single species depending on habitat conditions. Therefore, environmental plasticity should be considered an important factor shaping trap morphology alongside phylogenetic constraints and adaptive evolution, especially since there are over 260 known species of *Drosera*, which exhibit enormous diversity in terms of morphology and habitats [27,28,29,30].

It should be noted that the phenomenon of the occurrence of the aquatic form of *D. intermedia* has so far been observed only in post-glacial lakes with a layer formed by *Sphagnum*, which overgrows the water surface [20]. In Czech populations of *D. intermedia* that were flooded, plants died if a deeper flooding in colored water lasted for a long time of several weeks [Adamec L. personal information. 18.05.2026]. In the context of *D. intermedia*, of particular interest is *Drosera amazonica* Rivadavia, A. Fleischm. & Vicent., a species from the Amazon basin that has adapted to survive underwater during seasonal fluctuations in water level [31]. This species disperses its seeds through water (hydrochory). Unfortunately, there is no information on how prolonged flooding affects the carnivorous syndrome in *Drosera amazonica*.

In the context of how terrestrial carnivorous plants’ traps function, an interesting observation made by Popping et al. [32] is that the traps of *Dionaea muscipula* J.Ellis can operate in an aquatic environment.

Several limitations should nevertheless be acknowledged. The present study focused primarily on morphology and did not directly assess prey capture efficiency, digestive activity, mucilage chemistry, or trap biomechanics under aquatic conditions. Furthermore, it remains unclear whether the observed modifications are entirely environmentally induced or partially genetically fixed within submerged populations. An additional limitation is that multiple environmental variables differed among the investigated habitats. Therefore, although the observed morphological differences are associated with the submerged habitat, the present study cannot determine the relative contribution of submergence, light availability, substrate properties, temperature, pH, or other environmental factors. Future studies combining controlled experiments with multivariate statistical approaches (e.g., PCA, RDA, or GLM) would help disentangle the influence of individual environmental drivers on trap morphology in *Drosera intermedia*. Furthermore, integrating anatomy, biomechanics, physiology, and transcriptomics could clarify the mechanisms underlying trap modification in submerged plants.

## 4. Materials and Methods

### 4.1. Analysis of Environmental Parameters

Plant material of *Drosera intermedia* Hayne was collected in June 2024 from two sites: (1) peatlands surrounding Lake Moczadło within the Zaborski Landscape Park near Męcikał (Chojnice County, Pomeranian Voivodeship, 53°48′52.7″ N 17°38′27.6″ E; emergent wetland habitats, and submerged aquatic habitats) and (2) the shore of Lake Moczadło (53°48′51.2″ N 17°38′00.7″ E; sandy terrestrial habitats). At each site, environmental conditions were recorded, and the architectures of six individuals of the respective sundew species were measured at 5 localities (a total of 30 individuals were collected from each habitat type, although only 20 underwater specimens were collected due to the limited number of such plants). To this end, the plants were uprooted. All specimens from a given site were carefully laid out on a special, scaled mat and photographed to enable the measurement of architectural features using graphics software (CorelDRAW X6). Three typical individuals of each sundew species were preserved as herbarium specimens in the Herbarium Universitatis Gedanensis UGDA. Based on the photographs, the plant architecture was measured, including the number of living and dead leaves, leaf length, and the width and length of the leaf blade. The collection of *D. intermedia* and research in the Moczadło Lake Reserve were conducted under permits issued by the Regional Directorate for Environmental Protection in Gdańsk.

The environmental conditions were determined at each locality (6 for each habitat type, excluding aquatic habitats, which consisted of a single reservoir and were measured at only one location) based on the following:

- Photosynthetically active radiation (PAR) was measured using a LI-250 light meter (LI-COR, Lincoln, NE, USA) and then converted into a percentage of the light reaching a fully illuminated site;
- pH and temperature—using a WTW 320/SET1 pH meter (WTW, Xylem Analytics, Weilheim, Germany) and a SENTIX 97T measuring electrode (WTW, Xylem Analytics, Weilheim, Germany);
- Electrical conductivity—using a WTW Cond 3210 SET 2 conductivity meter (WTW, Xylem Analytics, Weilheim, Germany);
- Substrate moisture content—calculated as a percentage of the difference between the mass of fresh substrate and substrate dried to constant weight at 105 °C in a Binder FD115 laboratory oven (BINDER GmbH, Tuttlingen, Germany);
- The organic matter content in the substrate was calculated as a percentage of the difference in mass between the dry substrate and the substrate calcined in a SEL 96C muffle furnace (Czylok Sp. z o.o., Jastrzębie-Zdrój, Poland) at 550 °C for 5 h.

Chlorophyll fluorescence (Fv/Fm parameter) was measured for each of the 30 individuals from the habitat type (20 underwater specimens) using a Handy Pea fluorometer (Hansatech Ltd., Pentney, UK), as described by Aksmann et al., 2016 [33], always on the third leaf (fully developed and mature) counting from the shoot axis.

Leaves of *D. intermedia* for tentacles measurements were sampled from 10 individuals typical of each habitat, namely submerged, emergent, and sandy terrestrial sites, as in [20]. The leaves collected for this purpose were fully developed and mature (this was the 3rd or 4th leaf from the shoot apex). Leaves were fixed in 70% ethanol immediately after sampling and then stored at 4 °C. To prepare for scanning electron microscopy (SEM), the traps were fixed in 70% ethanol, later increased to 100% ethanol, and then transferred to acetone and dried using supercritical CO_2_. The material was then sputter-coated with gold and examined using a Hitachi S-4700 scanning electron microscope (Tokyo, Japan), which is housed at the Institute of Geological Sciences, Jagiellonian University, Kraków, Poland, or a Hitachi UHR FE-SEM SU 8010 microscope (Hitachi, Tokyo, Japan), which is housed at the Institute of Biology, Biotechnology and Environmental Protection, Faculty of Natural Sciences University of Silesia in Katowice.

### 4.2. Statistical Analysis Methods

From each habitat type, ten individuals were selected, and one mature leaf per plant was randomly sampled and prepared for microscopic examination in anhydrous glycerol. All emergences present on each leaf were measured. A total of 803 emergences were measured in the central leaf regions (>500 µm from the end of leaf blade margin) and 1445 in the marginal regions (<500 µm from the end of leaf blade margin). Morphometric measurements of tentacle length and head size were obtained using a Nikon Eclipse E400 microscope (Nikon, Japan) with digital imaging support. When tentacles required multisegmented measurements, segment lengths were summed manually.

Tentacles head shapes were calculated as length-to-width ratios. Emergence lengths and head dimensions from central and marginal leaf regions were compared across the three habitat types. Tentacles from the same leaf are not independent observations; they were used solely to calculate the mean values of the traits (tentacle length, tentacle head size, and tentacle head shape) for each leaf/individual. In the study, the leaf/individual plant was treated as a random factor; therefore, the mean values of the traits calculated for each leaf/individual were analyzed (10 for each habitat types). Statistical analyses were performed in STATISTICA 13.1. To determine differences between habitat types, the Kruskal–Wallis test (α = 0.05) was applied, and significant results were followed by Dunn’s post hoc test.

## 5. Conclusions

The aquatic environment significantly modifies trap morphology in the submerged form of *Drosera intermedia*. Submerged plants develop shorter tentacles and smaller glandular heads compared with emergent forms, while certain trap features, particularly the shape of marginal tentacle heads, remain relatively stable. These results may indicate substantial phenotypic plasticity of the carnivorous apparatus and suggest partial functional reorganization of the carnivorous syndrome under aquatic conditions. The persistence of modified traps in submerged plants demonstrates that carnivory may be retained despite strong environmental alteration, highlighting the remarkable ecological flexibility of *D. intermedia*.

## Figures and Tables

**Table 1:** Lengths of tentacles on the marginal and central parts of the leaf blades of *D. intermedia* depending on habitat type.

Habitat Type	N	Mean	SD	Median	Minimum	Maximum
Lengths of marginal tentacles [μm]						
Submerged	10	2057.827	258.3710	2116.381	1611.824	2440.074
Emergent	10	2759.839	221.3175	2765.329	2461.262	3224.579
Sand	10	2195.802	210.8400	2164.654	1934.668	2543.351
Lengths of central tentacles [μm]						
Submerged	10	625.254	115.8933	603.920	462.733	839.629
Emergent	10	805.383	59.8028	802.923	721.575	907.848
Sand	10	720.839	118.0592	745.110	499.540	903.631

**Table 2:** Results of the Dunn post hoc test for comparisons of emergence lengths on the marginal and central parts of leaf blades of *D. intermedia* between habitat types.

Lengths of Marginal Tentacles			
Comparison	Mean rank—group 1	Mean rank—group 2	*p*-value
Submerged vs. Emergent	9.000	25.200	0.001995
Submerged vs. Sand	9.000	12.300	0.22621
Emergent vs. Sand	25.200	12.300	0.31210
**Lengths of** **central tentacles**			
Comparison	Mean rank—group 1	Mean rank—group 2	*p*-value
Submerged vs. Emergent	9.000	25.200	0.000116
Submerged vs. Sand	9.000	12.300	1.000
Emergent vs. Sand	25.200	12.300	0.00315

**Table 3:** The shapes of tentacle heads on the marginal and central parts of blades of *D. intermedia* depending on habitat type.

Habitat Type	N	Mean	SD	Median	Minimum	Maximum
Shapes of marginal tentacle heads						
Submerged	10	2.092	0.1017	2.098	1.928	2.258
Emergent	10	2.063	0.0768	2.043	1.953	2.198
Sand	10	2.066	0.1529	2.085	1.826	2.266
Shapes of central tentacle heads						
Submerged	10	1.728	0.0911	1.715	1.638	1.921
Emergent	10	1.808	0.1074	1.763	1.696	1.996
Sand	10	1.805	0.1748	1.747	1.565	2.052

**Table 4:** Length of tentacle heads on the marginal and central parts of leaf blades of *D. intermedia* across habitat types.

Habitat Type	N	Mean	SD	Median	Minimum	Maximum
Length of marginal tentacle heads [μm]						
Submerged	10	244.367	22.4537	247.569	205.471	276.095
Emergent	10	282.114	9.3075	281.082	271.428	297.863
Sand	10	236.831	22.8620	234.100	195.744	277.226
Length of central tentacle heads [μm]						
Submerged	10	227.872	32.2758	227.786	181.669	268.254
Emergent	10	268.522	18.4006	268.031	238.616	292.981
Sand	10	227.813	20.8098	228.876	192.379	261.433

**Table 5:** Approximate *p*-values for multiple comparisons of the lengths of tentacle heads on the central parts of leaf blades of *D. intermedia* between habitat types. Analysis based on the Dunn test.

Lengths of Marginal Tentacle Heads			
Comparison	Mean rank—group 1	Mean rank—group 2	*p*-value
Submerged vs. Emergent	11.900	24.900	0.002880
Submerged vs. Sand	11.900	9.7000	1.0000
Emergent vs. Sand	24.900	9.7000	0.000339
**Lengths of** **central tentacle heads**			
Comparison	Mean rank—group 1	Mean rank—group 2	*p*-value
Submerged vs. Emergent	12.400	23.400	0.015618
Submerged vs. Sand	12.400	10.700	1.0000
Emergent vs. Sand	23.400	10.700	0.003769

**Table 6:** The width of tentacle heads on the marginal and central parts of leaf blades of *D. intermedia* across habitat types.

Habitat Type	N	Mean	SD	Median	Minimum	Maximum
Width of marginal tentacle heads [μm]						
Submerged	10	118.327	11.7341	123.662	98.333	133.030
Emergent	10	138.398	8.8032	139.227	125.765	154.084
Sand	10	117.494	10.6875	119.897	102.695	133.730
Width of central tentacle heads [μm]						
Submerged	10	133.411	18.4609	137.165	103.796	154.563
Emergent	10	150.212	15.5050	149.067	126.182	173.285
Sand	10	132.738	16.2984	133.046	109.526	167.108

**Table 7:** Approximate *p*-values for multiple comparisons of the widths of tentacle heads on the marginal parts of leaf blades of *Drosera intermedia* between habitat types. Analysis based on the Dunn test.

Widths of Marginal Tentacle Heads			
Comparison	Mean rank—group 1	Mean rank—group 2	*p*-value
Submerged vs. Emergent	11.400	24.500	0.002630
Submerged vs. Sand	11.400	10.6000	1.0000
Emergent vs. Sand	24.500	10.6000	0.001244

**Table 8:** Summary of morphological traits and habitat characterictics of *D. intermedia* populations growing in sandy habitats.

	N	Mean	SD	Median	Min	Max
Number of live leaves	30	29.80	12.424	31.00	10.00	57.00
Number of dead leaves	30	6.80	4.89	5.00	1.00	21.00
Length of leaves	30	2.53740	0.46455	2.5710	1.07116	3.1958
Leaf blade width [cm]	30	0.25937	0.03385	0.2599	0.16364	0.3202
Leaf blade length [cm]	30	0.66031	0.10320	0.6737	0.39170	0.8739
Leaf blade elongation	30	2.55401	0.31031	2.5216	1.99837	3.2248
pH	30	4.88	0.39	4.84	4.45	5.39
Conductivity [μS/cm]	30	36.06	24.067	27.30	16.50	80.20
Temperature [°C]	30	17.76	0.85	17.90	16.20	18.60
PAR [%]	30	64.24	44.55	100.00	10.60	100.00
Hydration [%]	30	27.28	13.70	26.10	9.90	47.30
Organic matter [%]	30	15.64	14.36	15.90	1.80	40.80
Fv/Fm	30	0.71483	0.04155	0.7130	0.56500	0.8000

**Table 9:** Summary of morphological traits and habitat characteristics for emergent populations of *D. intermedia*.

	N	Mean	SD	Median	Min	Max
Number of live leaves	30	18.67	9.60	16.50	7.00	43.00
Number of dead leaves	30	11.23	5.14	11.00	2.00	23.00
Length of leaves	30	3.34018	0.96627	3.1930	2.03281	5.4098
Leaf blade width [cm]	30	0.25485	0.03173	0.2547	0.18195	0.3241
Leaf blade length [cm]	30	0.83000	0.22032	0.7984	0.54004	1.3709
Leaf blade elongation	30	3.24020	0.66731	3.0310	2.42568	4.9388
pH	30	4.16	0.36	4.05	3.81	4.74
Conductivity [μS/cm]	30	34.80	20.19	26.30	18.40	71.80
Temperature [°C]	30	17.30	0.92	17.20	16.10	18.90
PAR [%]	30	96.16	7.81	100.00	80.80	100.00
Hydration [%]	30	97.14	2.54	96.70	93.90	100.00
Organic matter [%]	30	94.01	1.49	94.30	91.20	95.18
Fv/Fm	30	0.58763	0.13216	0.6140	0.24800	0.7650

**Table 10:** Summary of morphological traits and habitat characteristics for submerged populations of *D. intermedia*.

	N	Mean	SD	Median	Min	Max
Number of live leaves	20	21.15	11.89	17.00	10.00	56.00
Number of dead leaves	20	6.30	3.03	6.00	2.00	13.00
Length of leaves	20	3.2769	0.70798	3.4256	1.9503	4.3630
Leaf blade width [cm]	20	0.2354	0.05314	0.2318	0.1669	0.3856
Leaf blade length [cm]	20	0.5016	0.17304	0.4635	0.2671	0.9332
Leaf blade elongation	20	2.0890	0.28234	2.0661	1.5691	2.7487
pH	20	4.74	0.00	4.7400	4.74	4.74
Conductivity [μS/cm]	20	19.10	0.00	19.10	19.10	19.10
Temperature [°C]	20	18.90	0.00	18.90	18.90	18.90
PAR [%]	20	59.40	0.00	59.40	59.40	59.40
Hydration [%]	20	100.00	0.00	100.00	100.00	100.00
Organic matter [%]	20	94.20	0.00	94.20	94.20	94.20
Fv/Fm	20	0.6504	0.09564	0.6685	0.3790	0.7970

## Data Availability

The original contributions presented in this study are included in the article. Further inquiries can be directed to the corresponding author.

## References

[1] Darwin C. (1875). Insectivorous Plants.

[2] Fenner C.A. (1904). Beiträge zur Kenntnis der Anatomie, Entwicklungsgeschichte und Biologie der Laubblätter und Drüsen einiger Insektivoren. Flora.

[3] Lüttge U., Lange O.L., Nobel P.S., Osmond C.B., Ziegler H. (1983). Ecophysiology of carnivorous plants. Physiological Plant Ecology III.

[4] Juniper B.E., Robbins R.J., Joel D.M. (1989). The Carnivorous Plants.

[5] Slack A. (2000). Carnivorous Plants.

[6] Barthlott W., Porembski S., Seine R., Theisen I. (2007). The Curious World of Carnivorous Plants: A Comprehensive Guide to Their Biology and Cultivation.

[7] Król E., Płachno B.J., Adamec L., Stolarz M., Dziubińska H., Trębacz K. (2012). Quite a few reasons for calling carnivores “the most wonderful plants in the world”. Ann. Bot..

[8] Adamec L. (2013). Foliar mineral nutrient uptake in carnivorous plants: What do we know and what should we know?. Front. Plant Sci..

[9] Ellison A.M., Adamec L. (2018). Carnivorous Plants: Physiology, Ecology, and Evolution.

[10] Givnish T.J., Burkhardt E.L., Happel R.E., Weintraub J.D. (1984). Carnivory in the bromeliad *Brocchinia reducta* with a cost/benefit model for the general restriction of carnivorous plants to sunny, moist, nutrient-poor habitats. Am. Nat..

[11] Givnish T.J., Sparks K.W., Hunter S.J., Pavlovič A., Ellison A.M., Adamec L. (2018). Why are plants carnivorous? Cost/benefit analysis, whole-plant growth, and the context-specific advantages of botanical carnivory. Carnivorous Plants: Physiology, Ecology, and Evolution.

[12] Lloyd F.E. (1942). The Carnivorous Plants.

[13] Hartmeyer I., Hartmeyer S.R.H. (2006). Clandestine diversity: Snap-tentacles of the genus *Drosera*. Carniflora Aust..

[14] Poppinga S., Hartmeyer S.R., Masselter T., Hartmeyer I., Speck T. (2013). Trap diversity and evolution in the family Droseraceae. Plant Signal. Behav..

[15] Lichtscheidl I., Lancelle S., Weidinger M., Adlassnig W., Koller-Peroutka M., Bauer S., Krammer S., Hepler P.K. (2021). Gland cell responses to feeding in *Drosera capensis*, a carnivorous plant. Protoplasma.

[16] Adlassnig W., Koller-Peroutka M., Bauer S., Koshkin E., Lendl T., Lichtscheidl I.K. (2012). Endocytotic uptake of nutrients in carnivorous plants. Plant J..

[17] Ivesic C., Krammer S., Koller-Peroutka M., Laarouchi A., Gruber D., Lang I., Lichtscheidl I.K., Adlassnig W. (2023). Quantification of protein uptake by endocytosis in carnivorous Nepenthales. Plants.

[18] Ivesic C., Adlassnig W., Koller-Peroutka M., Kress L., Lang I. (2022). Snatching sundews—Analysis of tentacle movement in two species of *Drosera* in terms of response rate, response time, and speed of movement. Plants.

[19] Poppinga S., Hartmeyer S.R.H., Seidel R., Masselter T., Hartmeyer I., Speck T. (2012). Catapulting tentacles in a sticky carnivorous plant. PLoS ONE.

[20] Banaś K., Aksmann A., Płachno B.J., Kapusta M., Marciniak P., Ronowski R. (2024). Individual architecture and photosynthetic performance of the submerged form of *Drosera intermedia* Hayne. BMC Plant Biol..

[21] Ellison A.M., Gotelli N.J. (2002). Nitrogen availability alters the expression of carnivory in the northern pitcher plant, *Sarracenia purpurea*. Proc. Natl. Acad. Sci. USA.

[22] Green S., Green T.L., Heslop-Harrison Y. (1979). Seasonal heterophylly and leaf gland features in *Triphyophyllum* (Dioncophyllaceae), a new carnivorous plant genus. Bot. J. Linn. Soc..

[23] Winkelmann T., Bringmann G., Herwig A., Hedrich R. (2023). Carnivory on demand: Phosphorus deficiency induces glandular leaves in the African liana *Triphyophyllum peltatum*. New Phytol..

[24] Fukushima K., Narukawa H., Palfalvi G., Hasebe M. (2021). A discordance of seasonally covarying cues uncovers misregulated phenotypes in the heterophyllous pitcher plant *Cephalotus follicularis*. Proc. Biol. Sci..

[25] Whitewoods C.D., Gonçalves B., Cheng J., Cui M., Kennaway R., Lee K., Bushell C., Yu M., Piao C., Coen E. (2020). Evolution of carnivorous traps from planar leaves through simple shifts in gene expression. Science.

[26] Banaś K., Ronowski R., Marciniak P. (2023). Effects of Environmental Conditions on the Individual Architectures and Photosynthetic Performances of Three Species in *Drosera*. Int. J. Mol. Sci..

[27] Lowrie A., Robinson A., Nunn R., Rice B., Bourke G., Gibson R., McPherson S., Fleischmann A. (2017). Drosera of the World, Volume 1: Oceania.

[28] Lowrie A., Robinson A., Nunn R., Rice B., Bourke G., Gibson R., McPherson S., Fleischmann A. (2017). Drosera of the World, Volume 2: Oceania, Asia, Europe and North America.

[29] Lowrie A., Robinson A., Nunn R., Rice B., Bourke G., Gibson R., McPherson S., Fleischmann A. (2017). Drosera of the World, Volume 3: Latin America and Africa.

[30] Fleischmann A., Cross A.T., Gibson R., Gonella P.M., Dixon K.W., Ellison A.M., Adamec L. (2018). Systematics and evolution of Droseraceae. Carnivorous Plants: Physiology, Ecology, and Evolution.

[31] Rivadavia F., Vicentini A., Fleischmann A. (2009). A New Species of Sundew (*Drosera*, Droseraceae), with Water-Dispersed Seed, from the Floodplains of the Northern Amazon Basin, Brazil. Ecotropica.

[32] Poppinga S., Kampowski T., Metzger A., Speck O., Speck T. (2016). Comparative kinematical analyses of Venus flytrap (*Dionaea muscipula*) snap traps. Beilstein J. Nanotechnol..

[33] Aksmann A., Pokora W., Baścik-Remisiewicz A., Dettlaff-Pokora A., Tukaj Z. (2016). High hydrogen peroxide production and antioxidative enzymes expression in the *Chlamydomonas reinhardtii* cia3 mutant with an increased tolerance to cadmium and anthracene. Phycol. Res..

